# Establishing thresholds for meaningful within-individual change using longitudinal item response theory

**DOI:** 10.1007/s11136-022-03172-5

**Published:** 2022-07-23

**Authors:** Jakob Bue Bjorner, Berend Terluin, Andrew Trigg, Jinxiang Hu, Keri J. S. Brady, Pip Griffiths

**Affiliations:** 1 QualityMetric Incorporated, LLC, Johnston, RI USA; 2 University of Copenhagen, Copenhagen, Denmark; 3 Amsterdam University Medical Centers, Amsterdam, The Netherlands; 4 Adelphi Values, Bollington, UK; 5 University of Kansas Medical Center, Kansas City, KS USA; 6 Department of Health Law, Policy & Management, Boston University School of Public Health, Boston, USA; 7 IQVIA France, Paris, France

**Keywords:** Meaningful within-individual change, Minimal important change, Minimal important difference, Item response theory, Longitudinal studies, Interpretation of Patient-Reported Outcomes

## Abstract

**Purpose:**

Thresholds for meaningful within-individual change (MWIC) are useful for interpreting patient-reported outcome measures (PROM). Transition ratings (TR) have been recommended as anchors to establish MWIC. Traditional statistical methods for analyzing MWIC such as mean change analysis, receiver operating characteristic (ROC) analysis, and predictive modeling ignore problems of floor/ceiling effects and measurement error in the PROM scores and the TR item. We present a novel approach to MWIC estimation for multi-item scales using longitudinal item response theory (LIRT).

**Methods:**

A Graded Response LIRT model for baseline and follow-up PROM data was expanded to include a TR item measuring latent change. The LIRT threshold parameter for the TR established the MWIC threshold on the latent metric, from which the observed PROM score MWIC threshold was estimated. We compared the LIRT approach and traditional methods using an example data set with baseline and three follow-up assessments differing by magnitude of score improvement, variance of score improvement, and baseline-follow-up score correlation.

**Results:**

The LIRT model provided good fit to the data. LIRT estimates of observed PROM MWIC varied between 3 and 4 points score improvement. In contrast, results from traditional methods varied from 2 to 10 points—strongly associated with proportion of self-rated improvement. Best agreement between methods was seen when approximately 50% rated their health as improved.

**Conclusion:**

Results from traditional analyses of anchor-based MWIC are impacted by study conditions. LIRT constitutes a promising and more robust analytic approach to identifying thresholds for MWIC.

**Supplementary Information:**

The online version contains supplementary material available at 10.1007/s11136-022-03172-5.

## Introduction

Patient-Reported Outcome Measures (PROMs) bring the necessary patient perspective into the evaluation of treatment outcomes—assessing constructs such as pain, physical functioning, or depression. The PROM score (often the sum of responses to several items) provides an estimate of the construct at individual or group levels. When PROMs are administered longitudinally (e.g., pre- and post-treatment), score differences estimate individual or mean group change. A problem in interpreting PROM scores is that they lack intrinsic meaning [1]. It is difficult to tell if a given PROM change score (of e.g., 10 points on a 100 points scale) represents a substantial or meaningful change. To assist PROM users in the interpretation of change scores, the concept of the “minimal important change” (MIC, also denoted “minimal important difference”, “minimal clinically important difference” or “meaningful change threshold”) was introduced [2, 3]. Subsequent discussions have distinguished changes between groups, within groups, or within individuals [4, 5]. This paper considers thresholds for meaningful within-individual change (MWIC).

Methods to establish MIC and MWIC thresholds fall in two major categories: distribution-based and anchor-based methods [6, 7]. Anchor-based methods employ an external criterion (an anchor), often a patient-reported global rating of change, which we denote a transition rating (TR) [8]. We will focus on anchor-based methods, because they incorporate the patient’s perspective of meaningful change [9]. Both PROM improvement and deterioration may be of interest, but for the sake of simplicity, we will focus on MWIC thresholds for improvement. The methods are equally applicable to thresholds for deterioration.

Traditional statistical methods to estimate thresholds for MIC or MWIC based on TRs include the mean change method [9], the receiver operating characteristic (ROC) method [10], and the predictive modeling method [11]. These methods have a number of drawbacks. The mean change method simply takes the mean PROM change score in the group rating their perceived change as small but meaningful (e.g., “a little better”) as the MWIC. It is unclear whether this group average can serve as a threshold for MWIC, and it is typically higher than thresholds derived by other analyses [12]. Also, measurement error in the TR item may bias the selection of respondents with small but meaningful improvement. The ROC method seeks to identify the PROM change score threshold that optimally classifies patients into improved (e.g., indicating “a little better” or “much better” on the TR item) from not-improved (e.g., indicating “unchanged” or worse) patients. Often the threshold that minimizes the absolute difference between sensitivity and specificity is chosen as MWIC threshold, ensuring the least misclassification [13]. However, the ROC-based MWIC has been shown to be imprecise, due to measurement error in the PROM change score [11], and it is biased by the proportion of improved patients [14]. The predictive modeling method uses logistic regression modeling to identify the PROM change score that is equally likely to occur in the improved and not-improved groups (i.e., the PROM change score with a likelihood ratio of 1) [11, 15]. This approach assumes a linear effect of the PROM change score on the log-odds of TR response, which may not be realistic in case of floor or ceiling effects. The approach is also biased by the proportion of improved patients, although procedures have been proposed to adjust for this bias [14].

In this paper, we will introduce a new approach to estimate an MWIC, based on longitudinal item response theory (LIRT) [16]. Item response theory (IRT) refers to a group of mathematical models describing the relationship between the responses to the items of a questionnaire and the constructs the questionnaire is purported to measure [17, 18]. The constructs are modeled as latent (i.e., not directly observable) variables [19]. We will use a LIRT model to estimate an MWIC threshold based on responses to a TR item. First, we show how the MWIC threshold can be defined and estimated on the latent scale. Second, we use the LIRT model to estimate the MWIC for the observed PROM change score. Third, we discuss how to evaluate LIRT model assumptions. Fourth, we evaluate the new approach using the dataset provided for this special issue and we compare our LIRT-based MWIC results with the results of the traditional approaches.

## Methods

### Defining and estimating MWIC thresholds on the latent scale

**Fig. 1:**
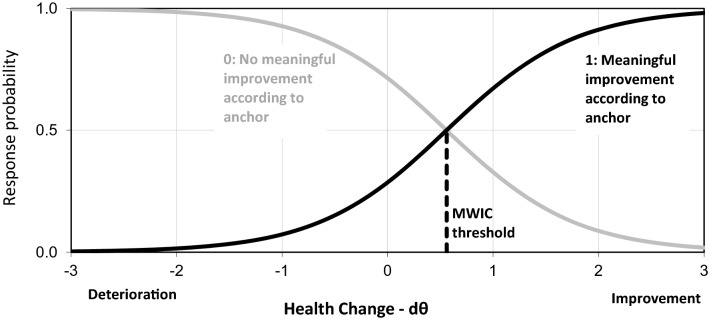
Probability of rating a health improvement as meaningful according to the IRT model for a transition rating item

We start by developing a statistical model for a situation where a set of items measuring a particular health construct has been administered at baseline (time 1) and follow-up (time 2). Also, a TR item assessing change in the same construct has been administered at time 2. We assume that patients, when asked to rate their change between two timepoints, perform an internal comparison of their perceived change with individual thresholds for, e.g., “meaningfully improved” [14, 20], which we denote “individual MWIC” [14]. Plausibly, individual MWICs are not the same for every person [14, 20]. The best we can do is to estimate the mean of the individual MWICs. For simplicity of discussion, we assume that responses to the TR item have been dichotomized into 1: meaningful improvement and 0: no meaningful improvement. In other words, we only consider one threshold, the one for meaningful improvement. However, the model can easily be extended to cover all the response categories on the TR item (please see Web Appendix). The IRT model for the TR item can be specified in the following way (see Web Appendix for details):

1 $$ln\left( {\frac{{P\left( {TR_{j} = 1} \right)}}{{P\left( {TR_{j} = 0} \right)}}} \right) = \alpha_{TR} \left( {d\theta_{j} - \beta_{TR} } \right),$$

where $ln\left( {\frac{{P\left( {TR_{j} = 1} \right)}}{{P\left( {TR_{j} = 0} \right)}}} \right)$ is the log-odds of person *j* scoring ‘1’ on the TR, $d\theta_{j}$ is the latent change from time 1 to time 2 for person *j* (i.e., $\theta_{2j}$–$\theta_{1j}$), $\beta_{TR}$ is the MWIC threshold, and $\alpha_{TR}$ is the so-called discrimination parameter reflecting the TR item’s measurement precision (see Web Appendix). This IRT model is illustrated in Fig. 1, which shows the probability of indicating a meaningful improvement on the TR item for different levels of latent health change. The MWIC threshold is the level of health improvement where the probability of answering 1 (meaningful improvement) equals the probability of 0 (no meaningful improvement), i.e., 50%. This threshold can be interpreted as representing the mean of the individual MWICs (see Web Appendix). The model in Fig. 1 has an MWIC threshold of 0.56.

It should be noted that the IRT model in Eq. 1 and Fig. 1 is a standard two-parameter IRT model except that the TR item is an indicator of the latent change ($\theta_{2j}$–$\theta_{1j}$) instead of the latent state ($\theta_{1j}$ or $\theta_{2j}$). To estimate the model, we can embed the IRT model for the TR item within an LIRT model.

**Fig. 2:**
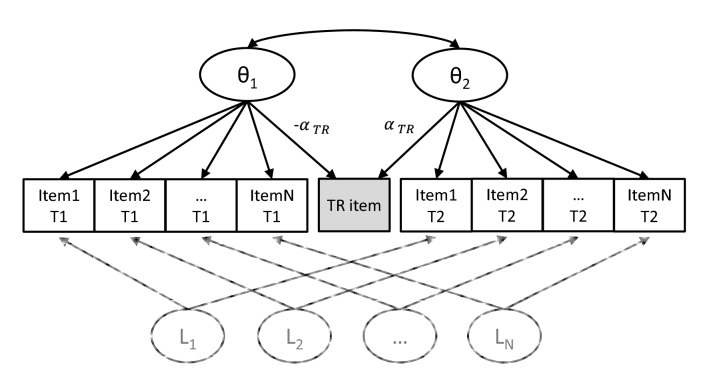
A longitudinal IRT model including a transition rating (TR) item

Such an LIRT model is illustrated in Fig. 2. The LIRT model is a special multidimensional IRT model in which the timepoints represent the dimensions of the model. By constraining the item characteristics to be the same across time, the latent mean and variance of the construct of interest can be estimated for each timepoint. This way, the latent change (i.e., $\theta_{2j}$–$\theta_{1j}$) of the construct between the timepoints can be estimated indirectly. The TR item, as an indicator of the latent change, should load on the latent states at both time 1 ($\theta_{1}$) and time 2 ($\theta_{2}$). Moreover, the TR item (being an indicator of the latent change) should be an indicator of time 1 as much as an indicator of time 2. Therefore, we impose the restriction that the discrimination parameter for $\theta_{1}$ is of the same magnitude but opposite sign as the discrimination parameter for $\theta_{2}$ (please see Web Appendix for justification). With this restriction, the item parameters for the TR item ($\alpha_{TR}$, $\beta_{TR}$, Eq. 1) can be estimated from the LIRT model. The bottom half of Fig. 2 (in gray) illustrates a possible model extension: it is plausible that responses to the same item at two different time points are locally dependent. This local dependence can be modeled by including a latent variable for each item capturing the local dependence across time—sometimes also called a two-tier model [21]. These LIRT models can be estimated with modern software for multidimensional IRT (e.g., Mplus [22] or the mirt package in R [23]), and $\beta_{TR}$ can be estimated directly (in Mplus) or indirectly (in mirt).

### Estimating MWIC thresholds on the observed PROM scale

For scales that are scored by IRT estimation of the latent score $\theta$ (e.g., scales from the PROMIS project), the MWIC threshold is provided by the $\beta_{TR}$ estimate. However, most PROMs are scored by calculating the sum of the items (which may be linearly transformed to a metric ranging from 0 to 100 or to a T-score metric). To estimate the MWIC for the observed PROM change score, we use a 4-step Monte-Carlo estimation procedure based on the parameters from the LIRT model: (1) Simulate the distribution of $\theta_{1}$ using the mean and variance from the above LIRT model (typically fixed at 0 and 1). (2) Calculate $\theta_{2}$ scores based on the assumption that all respondents had a latent improvement ($d\theta_{j}$) of exactly $\beta_{TR}$ (the MWIC threshold on the latent scale). (3) Simulate item responses at T1 and T2 based on $\theta_{1}$, $\theta_{2}$, and the estimated LIRT item parameters; calculate the “observed” PROM scores and PROM change scores. (4) Use the median PROM change score as the MWIC threshold for the observed PROM change score. We use the median rather than the mean since the distribution of PROM change scores may be skewed if the mean baseline score is close to the floor or the ceiling of the scale. However, we also present the mean PROM change score for comparison.

### Model assumptions and test of model fit

The described LIRT model makes five major assumptions that can all be tested: (1) At each time point, the IRT model should provide good fit to the PROM items. This assumption can be tested by standard IRT methods, e.g., by a generalization of the S-X^2^ test [24] to polytomous items. Misfit may lead to use of a more general IRT model (such as the nominal categories model [25]) for some items. (2) The PROM item parameters are assumed to be the same for time 1 and time 2 (i.e., no item response shift). This assumption can be tested for one item at the time using a likelihood ratio test comparing a model constraining the item parameters for that item over time with a model without these constraints. In case of significant difference for some items, the item parameters for these items can be allowed to differ over time. (3) The simplest form of the LIRT model assumes local item independence across time. This can be tested by comparing models with and without local dependence using a likelihood ratio test. The magnitude of local dependence can be evaluated by the discrimination parameters for the local dependence latent variables. Significant local dependence can be included in the model. (4) The discrimination parameter for the TR item on $\theta_{1}$ is assumed to be of the same magnitude but opposite sign as the discrimination parameter for the TR item on $\theta_{2}$. This can be tested by comparing models with and without constraints on the discrimination parameter for the TR item. Differences in the magnitude of the discrimination parameter for $\theta_{1}$ and $\theta_{2}$ can be caused by present state bias [26]. (5) Finally, we assume that the TR item measures change in the same construct that is measured by the PROM items and that the LIRT model provides good fit for the TR item. This may be evaluated by estimating the expected proportion of respondents indicating improvement (i.e., answering 1) on the TR item for different levels of the observed PROM change score. These expected proportions can be derived by simulation based on the estimated model parameters. These expected proportions can then be compared with the observed proportions of positive answers (similar to the approach of Orlando and Thissen [24]).

### Analysis of example dataset

The example data set was created for this special issue by simulation. The details of the simulations were unknown to the analyst while performing the analyses. The dataset contains 2000 subjects’ responses to 12 hypothetical items from the Simulated Disease Questionnaire (SDQ-12). Each item has four response categories. Items were originally scored such that high scores indicate poor health; we reverse-coded the original scores to be consistent with our discussions, so high scores indicate good health. Scores are available for four time points: baseline and follow-up 1, follow-up 2, and follow-up 3. Additionally, responses to a seven category TR item are available at follow-up 1, 2, and 3. For the current analyses, we have collapsed responses to 1: Much improved, Moderately improved, Minimally improved, and 0: No change, Minimally worse, Moderately worse, Much worse. Upon completion of the analyses, we learned that the true population value of the MWIC threshold (as simulated) was 0.5 $d\theta$, and the same across follow-up time points.

Our analyses established an MWIC threshold for changes from baseline to follow-up 1, 2, and 3, respectively. For each follow-up, we estimated MWIC on the latent metric (see Web Appendix for Mplus and mirt code) and evaluated model assumptions according to the procedures described above. Then, we derived MWIC estimates for the observed PROM scale and compared our results with traditional analyses: mean change, ROC, and adjusted predictive modeling [14]. To obtain 95% confidence intervals (CIs) for the LIRT MWIC estimates on the observed metric, we derived observed PROM MWIC estimates based on the lower and upper bound $\beta_{TR}$ values, holding all other parameters in the model constant. For mean change analysis, 95% CIs were estimated directly, while for ROC analyses and adjusted predictive modeling, 95% CIs were achieved by empirical bootstrap (1000 samples). For ROC analyses, we choose as MWIC threshold the value that minimized the absolute difference between sensitivity and specificity [13].

## Results

The top part of Table 1 shows descriptive information for each of the three follow-up time points. Based on the mean PROM change score, the sample had no significant mean change at follow-up 1, but had mean improvements of 0.19 SD at follow-up 2, and 0.58 SD at follow-up 3. Similarly, the percentage of respondents, who themselves reported that they had improved, increased over time from 34.5% at follow-up 1, to 51.1% at follow-up 2, and 72.0% at follow-up 3. The polychoric correlations between the TR item and the PROM change score were between 0.57 and 0.61; far above the threshold 0.30, which is often used to assess whether a TR item is sufficiently associated with PROM score change to make MWIC estimation possible [27].

**Table 1:** Descriptive statistics and parameter estimates from analysis of MWIC

	Follow-up 1		Follow-up 2		Follow-up 3	
	Est	(95% CI)	Est	(95% CI)	Est	(95% CI)
*Descriptive information*						
Mean PROM change score (raw score)	−0.20	(−0.56: 0.17)	3.45	(2.99: 3.90)	10.24	(9.79: 10.7)
Mean PROM change score (ES)	−0.01	(−0.03: 0.01)	0.19	(0.17: 0.22)	0.58	(0.55: 0.60)
% improved	34.5%	(32.4%: 36.5%)	51.1%	(48.9%: 53.3%)	72.0%	(70.0%: 73.9%)
TR*PROM change score polychoric correlation	0.57	(0.53: 0.61)	0.58	(0.54: 0.62)	0.61	(0.57: 0.65)
*LIRT parameter estimates*						
$\theta_{1}$ mean^1^	0		0		0	
$\theta_{1}$ SD^1^	1		1		1	
$\theta_{2}$ mean/$d\theta$ mean^2^	−0.02	(−0.08: 0.03)	0.57	(0.50: 0.65)	1.95	(1.84: 2.07)
$\theta_{2}$ SD	1.20	(1.15: 1.26)	1.45	(1.38: 1.53)	1.70	(1.61: 1.80)
$d\theta$ SD	1.03	(0.98: 1.08)	1.47	(1.40: 1.54)	1.81	(1.72: 1.91)
$\theta_{1}$*$\theta_{2}$ Correlation	0.58	(0.54: 0.61)	0.33	(0.28: 0.37)	0.18	(0.13: 0.23)
$\alpha_{TR}$	1.63	(1.41: 1.86)	0.98	(0.86: 1.10)	0.94	(0.83: 1.06)
$\beta_{TR}$	0.56	(0.48: 0.65)	0.50	(0.39: 0.61)	0.46	(0.31: 0.61)
*MWIC estimates (PROM score metric) by different methods*						
LIRT (Median)^3^	4	(3: 4)	3	(3: 4)	3	(2: 4)
LIRT (Mean)	3.78	(3.31: 4.44)	3.44	(2.61: 4.29)	3.20	(1.90: 4.46)
Mean change^4^	2.74	(1.87: 3.61)	2.59^5^	(1.44: 3.73)	7.81	(6.37: 9.25)
MWIC–ROC analyses^3^	2	(0: 2)	4	(3: 5)	10	(8: 10)
MWIC–Adjusted predictive model^4^	1.78	(1.40: 2.16)	3.30	(2.86: 3.72)	6.71	(6.17: 7.18)
*MWIC estimates by different methods – effect sizes and (% of score range)*						
LIRT (Median)	0.52	(11%)	0.39	(8%)	0.39	(8%)
LIRT (Mean)	0.49	(11%)	0.44	(10%)	0.41	(9%)
Mean change	0.35	(8%)	0.33^5^	(7%)	1.01	(22%)
MWIC–ROC analyses	0.26	(6%)	0.52	(11%)	1.29	(28%)
MWIC–Adjusted predictive model	0.23	(5%)	0.43	(9%)	0.87	(19%)

PROM change score: Difference in PROM score from baseline to specified follow-up timepoint. *ES*, Effect size. *TR*, Transition Rating. *LIRT*, Longitudinal item response theory. $\theta_{1}$: Latent score at baseline. $\theta_{2}$: Latent score at specified follow-up timepoint. $\alpha_{TR}$: Discrimination parameter for the transition rating item. $\beta_{TR}$: Threshold parameter for the transition rating item. MWIC: Meaningful within-individual change. ^1^For model identification, $\theta_{1}$ mean is fixed at 0 and $\theta_{1}$ SD is fixed at 1. ^2^Since $\theta_{1}$ mean = 0, $d\theta$ mean is equal to $\theta_{2}$ mean. ^3^MWIC estimates based on LIRT (median approach) or ROC analyses take only whole numbers, since they are based on values observed in the data set. ^4^Estimates based on mean change analyses or predictive modeling assume continuous measurement and can therefore take decimal values. ^5^Estimate is questionable, since the mean score change in the “no-change” group is larger (2.76) than in the meaningful change group.

The next section of Table 1 presents parameter estimates from LIRT modeling of each of the 3 follow-up times compared to baseline. Estimates of the mean $\theta_{2}$ values (equal to mean $d\theta$ since mean of $\theta_{1}$ is zero) supported the results of analyses of PROM change scores that no significant mean improvement was seen at follow-up 1, but increasing improvements were seen at follow-up 2 and 3. The $\theta_{2}$ SD estimates ranged from 1.2 at follow-up 1 to 1.7 at follow-up 3. Even larger increases were seen in the $d\theta$ SD estimates (which were calculated from $\theta_{1}$ SD, $\theta_{2}$ SD, and the $\theta_{1}$*$\theta_{2}$ correlation). The baseline*follow-up correlation decreased from 0.58 (at follow-up 1) to 0.18 (at follow-up 3). The TR item discrimination parameter varied between 0.94 (follow-up 3) and 1.63 (follow-up 1). Finally, the TR item threshold parameter estimates were close to the true value of 0.5, varying between 0.46 (follow-up 3) and 0.56 (follow-up 1). Figure 1 in the introduction illustrates the LIRT model for the TR item at follow-up 1 (i.e., $\alpha_{TR}$ = 1.63 and $\beta_{TR}$ = 0.56).

In tests of model fit at each time point, the generalized S-X^2^ item-level fit tests did not show significant misfit. Out of 48 item-level fit test (12 items at 4 time points), the lowest P value was 0.027 and only two P values were below 0.05. We did not find any indication of significant item response shift. Modeling of local item dependence across time did not improve model fit at any follow-up time point and was therefore not included in the final models. Equality of the discrimination parameters for the TR item on $\theta_{1}$ and $\theta_{2}$ was supported at all three follow-up times. The largest difference was found at follow-up time 3 where separate estimation yielded $\alpha_{TR1}$ = -1.05, $\alpha_{TR2}$ = 0.92 (likelihood ratio test for significant difference = 3.37, DF = 1, P = 0.066).

**Fig. 3:**
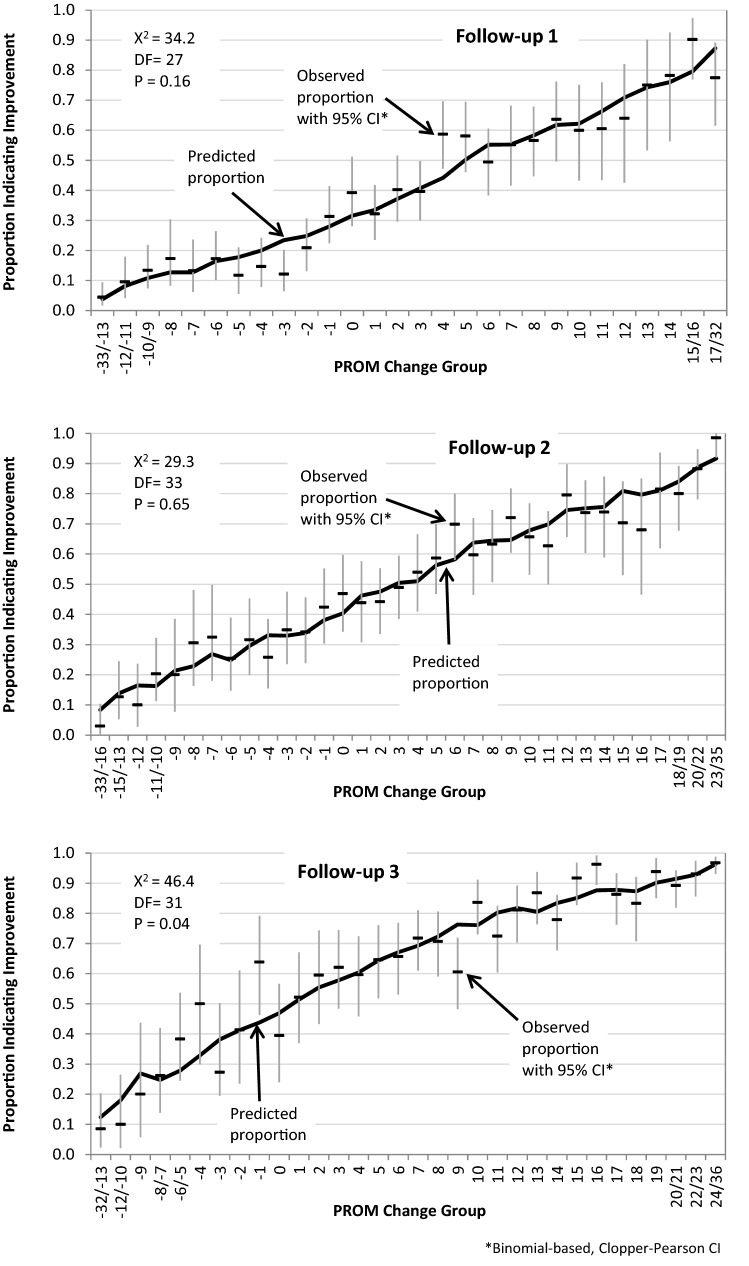
Testing the assumption of transition rating (TR) item fit: Plots of expected and observed proportions of respondents with different PROM change levels indicating improvement on the transition rating item. Full line: expected proportions of self-rated improvement. Dots with vertical lines: observed proportions with 95% CI according to a binomial model [31]. PROM change scores are collapsed at the extremes so that all expected cell frequencies are > 5

Figure 3 shows plots of expected and observed proportions of self-rated improvement along with the S-X^2^ test of fit, which was used to assess TR item fit. The model fit was acceptable at each follow-up time. Although the P value is 0.04 at follow-up 3, there were no indications of systematic departures from the predicted model.

**Fig. 4:**
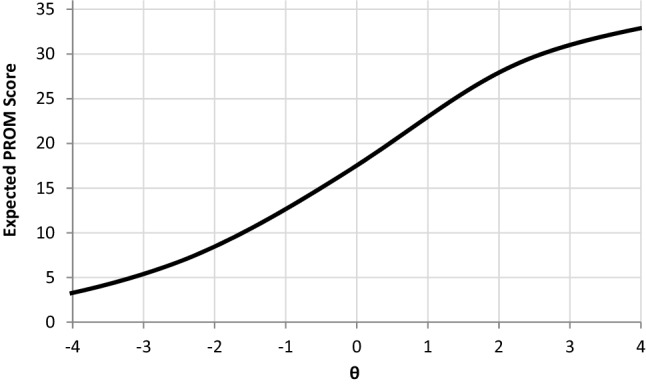
Plots of expected scores on the PROM across levels of θ. The expected score function is the same for time 1 and time 2

Figure 4 shows that in the θ range from -1 to 1.5, the expected PROM score function is almost linear. At baseline (where mean of $\theta_{1}$ = 0 and SD $\theta_{1}$ = 1), very few respondents were close to the floor or ceiling of the scale (indicated by a flat expected PROM score function).

Estimates of MWIC based on LIRT varied between 3 and 4 points score improvement in the PROM scale metric (equivalent to effect sizes of 0.39 and 0.52) (Table 1). Estimates of MWIC based on mean change, ROC analyses, and predictive modeling were lower than the LIRT-based estimate for follow-up 1 (range 1.78–2.74) but higher than the LIRT-based estimate for follow-up 3 (range 6.71 to 10). At follow-up 2, all four methods seemed to agree on an MWIC estimate close to 3, but the estimate from the mean change analysis cannot be trusted since the mean PROM score change was larger in the group indicating ‘no-change’ on the TR item than in the group indicating ‘minimally improved.’

## Discussion

We introduced a new method to estimate the MWIC of a PROM, based on longitudinal IRT. This method was compared to traditional methods using a simulated data set of PROM and TR item responses. Whereas the new method identified MWIC values between 3 and 4 across the three follow-up points, the MWIC estimates derived from traditional methods showed much more variability, and bias related to the proportion of improved patients. The convergence of results at follow-up 2 is no coincidence as Terluin et al. [14] showed that if the proportion improved is 50%, the MWIC equals the mean PROM change score (provided the PROM scores are normally distributed). At follow-up 2, the proportion improved is 51.1% (i.e., close to 50%) and the mean PROM change score is 3.45. The MWIC determined by the LIRT method is 3.44 (mean method) and the predictive modeling MWIC is 3.30. Apparently, the true MWIC in terms of the PROM change score (corresponding to 0.5 $d\theta$) is in the range of 3.30–3.45. At follow-up 1 (proportion improved 34.5%), the LIRT method slightly overestimated the MWIC (but the difference was not significant), whereas the other methods underestimated the true MWIC. Conversely, at follow-up 3 (proportion improved 72.0%), the LIRT method slightly underestimated the MWIC (not significant), whereas the other methods overestimated the true MWIC.

It is well known that the ROC-based MWIC is biased by the proportion improved [14, 28]. The adjusted MWIC is supposed to adjust for this bias [14], but the present findings suggest that the adjusted MWIC is also biased by the proportion improved. The mean change MWIC appeared also to be biased by the proportion improved, a finding that has not been described before. We hypothesize that the most likely reason for the bias of traditional methods is that they do not adequately handle measurement error in the TR item and in the PROM change score. Measurement error in the TR item implies that a proportion of persons who rate themselves as improved would not provide the same rating if asked again. If true improvement (defined as $d\theta \ge \beta_{TR}$) only occurs in a small part of the sample (notably below 50%), a large proportion of those who rate themselves as improved provide this rating due to measurement error, thus the TR item overestimates the true proportion of improved respondents. The persons providing a TR rating of “Minimally improved” due to measurement error is likely to have a low PROM change score, thus biasing the MWIC threshold estimate of traditional methods downwards. In contrast, if the proportion of true improvement is large (notably above 50%), measurement error will cause TR to underestimate the true proportion of improved persons, thus biasing the MWIC threshold estimate upwards. This is a problem for techniques such as mean change, ROC analyses, and predictive modeling, which assume that the TR item is a gold standard without error. Recent work has provided a way to adjust responder analyses for measurement error in the change score [29]. This approach assumes that the TR item is without measurement error and that the sensitivity and specificity of the TR item are generalizable across studies.

## Advantages of the new method

*One* of the advantages of our suggested approach is that the expanded LIRT model can handle both measurement error in the TR item and in the PROM change score, since the LIRT method models the error-free latent variables underlying the PROM item and TR item responses. While TR item measurement error makes the MWIC less precise, the estimate will not be biased when using the LIRT approach, and the reliability of the TR item can be estimated from the model*.* For follow-up 1, the $\alpha_{TR}$ parameter of 1.63 implies an item reliability of 0.49 (see Web Appendix). Similarly, while the PROM change score will have measurement error, the independent variable in our model, $d\theta$, is not subject to error if the model is correctly specified.

## Limitations and future research

While the LIRT model provides a probability of rating ‘improved’ for each level of change, the prediction is uncertain for the individual patient. Our results only suggest that an individual patient with an observed score improvement at or above the MWIC threshold has a 50% or better chance of rating his/her improvement as meaningful. Three factors contribute to this lack of certainty: 1. Persons may differ in their individual threshold for MWIC [14]. Thus, the MWIC threshold estimated by the model can be seen as the mean of individual thresholds (see Web Appendix). 2. There may be individual differences in the way the TR item is interpreted and answered (what we would typically call measurement error) due to e.g., individual differences in interpretation of wording, context effects, or recall bias. These two factors cause the reliability of the TR item to be less than 1—often below 0.5 as in the current study. 3. A further source of random variation is the measurement error of the PROM change score as a measure of the true change ($d\theta$). Further research is needed to derive a patient-specific probability of evaluating his or her score change as meaningful. As discussed above for group level interpretation, if a large proportion of a group (notably above 50%) have $d\theta \ge \beta_{TR}$, measurement uncertainty will cause TR to underestimate this proportion. Similarly, if only a small proportion (notably below 50%) have $d\theta \ge \beta_{TR}$, measurement uncertainty will cause TR to overestimate this proportion. Thus, the proportion of persons with true change above the MWIC threshold will not be equal to the proportion of persons indicating meaningful change on a TR item. Both proportions can be estimated through LIRT modeling, but this requires further work.

The LIRT model requires several assumptions to be met. As we have shown, these assumptions can be evaluated and the LIRT model can often be revised to accommodate misfit. An important assumption is that the transition rating is a valid indicator of the latent change in the construct measured by the PROM. This can be tested by evaluating the item fit and by looking at the magnitude of the transition rating discrimination parameter. However, some TR validity problems may not be easily detected: e.g., social desirability bias or impact of other questions (order effects). These potential sources of error are general to all analytic methods and emphasize the wisdom of using multiple anchors and methods to establish a threshold for MWIC.

We have assumed that the discrimination parameter for the TR item on $\theta_{1}$ is of the same magnitude but opposite sign as the discrimination parameter for the TR item on $\theta_{2}$. However, as discussed above, transition ratings might be more affected by the present (i.e., follow-up) state than the change. Then, we might find that the discrimination parameter for the TR item on $\theta_{2}$ would be numerically larger than the discrimination parameter for the TR item on $\theta_{1}$. More work is needed to understand the best approach to MWIC estimation in this situation.

The translation from a theta change MWIC to a PROM scale change MWIC is not straightforward as the relationship between the theta and the PROM scale score is non-linear (Fig. 4). In this paper, we used a Monte-Carlo approach and chose the median PROM scale change for subjects who improved by exactly the MWIC on the latent scale. An alternative choice would be taking the mean of the PROM change score instead of the median. The approaches agree for the current analyses (see Table 1), since very few observations are at the floor or the ceiling and the change score distribution is fairly symmetrical. However, if baseline samples have highly skewed PROM scores and floor or ceiling effects, the approach to transforming MWIC threshold from the latent scale to the PROM change scale may matter more. Further work is needed to establish the best way to translate the theta change MWIC into a PROM scale change MWIC in case of floor or ceiling effects. Model extensions incorporating censoring may be a way to address floor or ceiling effects [30].

## Conclusions

We have presented a new approach to estimating MWIC based on TR items. This approach, using LIRT, gave consistent results in the data sets provided for this special issue. In contrast, results from traditional analyses of anchor-based MWIC were impacted by study conditions. Thus, LIRT constitutes a promising and more robust analytic approach to identifying thresholds for MWIC in multi-item scales.

## Supplementary Information

Below is the link to the electronic supplementary material.

Supplementary file1 (DOCX 74 KB)

## Data Availability

Data available through Journal Website.

## Code availability

Code available through Journal Website.
