# Supplementary material for: Establishing thresholds for meaningful within-individual change using longitudinal item response theory
Source: Qual Life Res. 2022 Jul 23;32(5):1267–76. doi: 10.1007/s11136-022-03172-5 (PMC10123029; doi:10.1007/s11136-022-03172-5)
Supplement: Supplementary file 1 — Supplementary file1 (DOCX 74 KB) [file 11136_2022_3172_MOESM1_ESM.docx]

**Web Appendix for:**

Bjorner JB, Terluin B, Trigg A, Jinxiang H, Brady KJS, Griffiths P. Establishing thresholds for meaningful within-individual change using longitudinal item response theory. Quality of Life Research 2022.

1. Statistical model
2. Mplus code example for longitudinal item response theory analysis
3. mirt code example for longitudinal item response theory analysis
4. Comparison of models for dichotomous and ordinal categorical transition ratings

## 1. Statistical model

Assume that a set of items measuring a particular health construct has been administered at baseline (time 1) and follow-up (time 2). Also, a transition rating (TR) item covering the same construct (scored 1: meaningful improvement and 0: no meaningful improvement) has been administered at time 2. The score for person *j* on item *i* at time 1 is labeled $X_{ij1}$, the score at time 2 is labeled $X_{ij2}$. The score on the TR item is labeled ${TR}_{j}$. The latent health score for person *j* at time 1 is labeled $\theta_{j1}$, the latent score at time 2 is labeled $\theta_{j2}$, and the latent score change from baseline to follow-up is labeled $d\theta_{j}$.

A simple model for MWIC would be a threshold value $\beta_{TR}$ indicating the minimal level of change that is deemed important by patients. So

${{TR}_{j}^{*}=\rho_{TR} d\theta}_{j}+\epsilon_{j}$

${TR}_{j}^{*}\geq\beta_{TR} \Rightarrow{TR}_{j}=1, {TR}_{j}^{*}<\beta_{TR} \Rightarrow{TR}_{j}=0$ (1)

Where ${TR}_{j}^{*}$ is a latent formulation of the TR on an underlying continuous scale. This model includes a random component $\epsilon_{j}$ (with a mean of 0) reflecting measurement error in the TR item. An additional contributor to $\epsilon_{j}$could be between-individual differences in the threshold for important change. Assuming such between-individual differences, $\beta_{TR}$ should be interpreted as the mean of the individual thresholds for important change. Both measurement error and between-individual differences in thresholds for MWIC may contributed to $\epsilon_{j}$; the model does not distinguish between the two contributions. $\rho_{TR}$ is a scaling constant. If ${\rho_{TR}}^{2}+{var}_{\epsilon_{j}}=1$, then ${\rho_{TR}}^{2}$ can be interpreted as the reliability of the TR item.

If $\epsilon_{j}$ has a normal distribution, the model above implies a normal-ogive IRT model [1, 2]. If $\epsilon_{j}$ has a logistic distribution, the model above implies a logistic IRT model. Normal-ogive and logistic IRT models are very similar [3]. This paper uses the logistic IRT model which we have found to provide more robust results for MWIC estimation.

The logistic IRT model for the TR item can be specified in the following way:

$P\left( {TR}_{j}=1 \right)=\frac{exp\left( \alpha_{TR}\left( {d\theta}_{j}-\beta_{TR} \right) \right)}{1+exp\left( \alpha_{TR}\left( {d\theta}_{j}-\beta_{TR} \right) \right)}$ (2)

or, for simplicity [4]

$ln\left( \frac{P\left( {TR}_{j}=1 \right)}{P\left( {TR}_{j}=0 \right)} \right)=\alpha_{TR}\left( {d\theta}_{j}-\beta_{TR} \right)$ (3)

where $\beta_{TR}$ is the MWIC threshold and $\alpha_{TR}$ is a so-called discrimination parameter reflecting the ability of the TR item to distinguish between large and small health changes. $\alpha_{TR}$ is inversely related to the variance of the random component $\epsilon_{j}$ in equation (1). For a logistic model, $\alpha_{TR}= 1.7\frac{\rho_{TR}}{\sqrt{{var}_{\epsilon_{j}}}}$ [1, 3].

To estimate the model, we need more indicators of health change. For this purpose, equation (3) can be rearranged the following way:

$ln\left( \frac{P\left( {TR}_{j}=1 \right)}{P\left( {TR}_{j}=0 \right)} \right)=\alpha_{TR}\left( d\theta_{j}-\beta_{TR} \right)=\alpha_{TR}\left( \theta_{j2}-\theta_{j1}-\beta_{TR} \right)={\alpha_{TR}\theta}_{j2}-{\alpha_{TR}\theta}_{j1}+\lambda_{TR}$ (4)

Here, the response on the transition rating item is modeled as a function of the latent scores at baseline and follow-up. Equation 4 demonstrates the rationale for imposing the restriction that the discrimination parameter for $\theta_{j1}$ is of the same magnitude but opposite sign as the discrimination parameter for $\theta_{j2}$. The intercept parameter $\lambda_{TR}={-\alpha}_{TR}\beta_{TR}$ is often used in multidimensional IRT models. $\theta_{j1}$ and $\theta_{j2}$ can be further characterized by including IRT models for the PROM items at baseline and follow-up. Using the Graded Response IRT model [5], these models can be written as:

$ln\left( \frac{P\left( X_{ij1}\geq c \right)}{P\left( X_{ij1}<c \right)} \right)=\alpha_{i}\theta_{j1}{+ \lambda}_{ic}$ for time 1, and (5)

$ln\left( \frac{P\left( X_{ij2}\geq c \right)}{P\left( X_{ij2}<c \right)} \right)=\alpha_{i}\theta_{j2}{+ \lambda}_{ic}$ for time 2 (6)

where $\alpha_{i}$ is the discrimination parameter for item *i* and $\lambda_{ic}$ is the intercept parameter for category *c* of item *i*. Item parameters ($\alpha_{i}$ and $L_{ic}$) are assumed to be identical for time 1 and time 2, but this assumption can be relaxed for some items. The model described by equations 4-6 can be estimated by software for multidimensional IRT, such as Mplus or the R package mirt. In this paper, the LIRT models were fitted using maximum likelihood estimation. To identify the model, the$\theta$variance at time 1 (${var}_{\theta_{j1}}$) is usually set to 1.

If ${var}_{{d\theta}_{j}}$=1 then $\alpha_{TR}= 1.7\frac{\rho_{TR}}{\sqrt{1-{\rho_{TR}}^{2}}} \overset{\Leftrightarrow}{} {\rho_{TR}}^{2}= \frac{{\alpha_{TR}}^{2}}{{1.7}^{2}+{\alpha_{TR}}^{2}}$

Thus, at follow-up 1, the $\alpha_{TR}$parameter of 1.63 and $d\theta$ SD of 1.03 implies a TR item reliability of 0.49. This is in line with empirical results on the reliability of the TR item [6].

References

1. Muthen, B. O., & Christoffersson, A. (1981). Simultaneous Factor Analysis of Dichotomous Variables in Several Groups. *Psychometrika,* 46, 407–419.

2. Takane, Y., & Leeuw, J. de (1987). On The Relationship Between Item Response Theory And Factor Analysis Of Discretized Variables. *Psychometrika,* 52(3), 393–408.

3. Camilli, G. (1994). Origin of the scaling constant d=1.7 in item response theory. *Journal of Educational and Behavioral Statistics,* 19, 293–295.

4. Bjorner, J. B., Chang, C. H., Thissen, D., & Reeve, B. B. (2007). Developing tailored instruments: item banking and computerized adaptive assessment. *Qual.Life Res.,* 16 Suppl 1, 95–108.

5. Samejima, F. (1997). Graded response model. In W. van der Linden & R. Hambleton (Eds.), *Handbook of Modern Item Response Theory* (pp. 85–100). Berlin: Springer.

6. Griffiths, P., Terluin, B., Trigg, A., Schuller, W., & Bjorner, J. B. (2021). A confirmatory factor analysis approach was found to accurately estimate the reliability of transition ratings. *Journal of Clinical Epidemiology,* 141, 36–45. doi:10.1016/j.jclinepi.2021.08.029.

## 2. Mplus code example for longitudinal item response theory analysis

## TITLE: Mplus MWIC analysis through LIRT

## Comparing Baseline (T1) and first followup (T2)

##

## DATA: FILE IS SDQ_t2.dat;

## FORMAT IS 7X,1I2,12I1,12I1;

## TYPE IS INDIVIDUAL;

##

## VARIABLE: NAMES ARE anchor

## T1_It01 - T1_It12

## T2_It01 - T2_It12; ! Defining variable names;

## CATEGORICAL ARE anchor

## T1_It01 - T1_It12

## T2_It01 - T2_It12; ! Defining variable to be categorical;

##

## MODEL: T1 BY T1_It01* T1_It02-T1_It12 (1-12); ! Defining the IRT model discrimination at T1;

## T1 BY anchor* (a1); ! Defining the anchor item discrimination at T1;

## T2 BY T2_It01* T2_It02-T2_It12 (1-12); ! T2 IRT model - discrimination constrained to the same value as at T1;

## T2 BY anchor* (a2); ! Defining the anchor item discrimination at T2;

##

## T1@1; ! Fixing theta1 variance to 1 in order to identify the model;

## T2* (rho2); ! Letting theta2 variance be freely estimated and referred to as rho2;

## [T2*]; ! Letting theta2 mean be freely estimated;

## T1 with T2 (COVAR); ! The covariance between theta1 and theta2 is referred to as COVAR;

## [ANCHOR$1] (Lambda); ! The lambda parameter for the anchor item is referred to as Lambda;

## [T1_It01$1] (13); ! The next many lines constrain the Lambda parameters to be equal between T1 and T2;

## [T2_It01$1] (13);

## [T1_It01$2] (14);

## [T2_It01$2] (14);

## [T1_It01$3] (15);

## [T2_It01$3] (15);

## [T1_It02$1] (16);

## [T2_It02$1] (16);

## [T1_It02$2] (17);

## [T2_It02$2] (17);

## [T1_It02$3] (18);

## [T2_It02$3] (18);

## [T1_It03$1] (19);

## [T2_It03$1] (19);

## [T1_It03$2] (20);

## [T2_It03$2] (20);

## [T1_It03$3] (21);

## [T2_It03$3] (21);

## [T1_It04$1] (22);

## [T2_It04$1] (22);

## [T1_It04$2] (23);

## [T2_It04$2] (23);

## [T1_It04$3] (24);

## [T2_It04$3] (24);

## [T1_It05$1] (25);

## [T2_It05$1] (25);

## [T1_It05$2] (26);

## [T2_It05$2] (26);

## [T1_It05$3] (27);

## [T2_It05$3] (27);

## [T1_It06$1] (28);

## [T2_It06$1] (28);

## [T1_It06$2] (29);

## [T2_It06$2] (29);

## [T1_It06$3] (30);

## [T2_It06$3] (30);

## [T1_It07$1] (31);

## [T2_It07$1] (31);

## [T1_It07$2] (32);

## [T2_It07$2] (32);

## [T1_It07$3] (33);

## [T2_It07$3] (33);

## [T1_It08$1] (34);

## [T2_It08$1] (34);

## [T1_It08$2] (35);

## [T2_It08$2] (35);

## [T1_It08$3] (36);

## [T2_It08$3] (36);

## [T1_It09$1] (37);

## [T2_It09$1] (37);

## [T1_It09$2] (38);

## [T2_It09$2] (38);

## [T1_It09$3] (39);

## [T2_It09$3] (39);

## [T1_It10$1] (40);

## [T2_It10$1] (40);

## [T1_It10$2] (41);

## [T2_It10$2] (41);

## [T1_It10$3] (42);

## [T2_It10$3] (42);

## [T1_It11$1] (43);

## [T2_It11$1] (43);

## [T1_It11$2] (44);

## [T2_It11$2] (44);

## [T1_It11$3] (45);

## [T2_It11$3] (45);

## [T1_It12$1] (46);

## [T2_It12$1] (46);

## [T1_It12$2] (47);

## [T2_It12$2] (47);

## [T1_It12$3] (48);

## [T2_It12$3] (48);

## MODEL CONSTRAINT: a2 = -a1; ! The anchor discrimination parameter for T2 is constrained to be euqual to T1 but of opposite sign;

## NEW (Beta); ! A new parameter Beta is named - this is the MWIC threshold;

## Beta = Lambda/a2; ! Beta is calculated from two other parameter estimates;

## NEW (Corr); ! A new parameter Corr is named - this is the T1*T2 correlation;

## Corr = covar/(1*SQRT(rho2)); ! The correlation is estimated as the covariance divided by the T1 and T2 SDs;

## NEW (dT_SD); ! A new parameter dT_SD is named - this is the standard deviation of the latent change;

## dT_SD = SQRT(1 + rho2 - 2*covar); ! dT_SD is calculated from the T1 and T2 variances and their covariance;

## NEW (T2SD); ! A new parameter T2SD is named - this is the standard deviation of theta2;

## T2SD = SQRT(rho2); ! The standard deviation of theta2 is calculated;

##

## ANALYSIS: ESTIMATOR IS ML; ! Specifies an IRT model and not a SEM model;

## 3. mirt code example for longitudinal item response theory analysis

## ##### IRT MIC ANALYSIS OF ISOQOL SDQ-12 dataset

## rm(list=ls(all=TRUE)) # Clean the working space

## # library(lavaan)

## library(mirt)

## library(ggplot2)

## library(pROC)

## # library(psych)

## N.mic <- 20000 # Sample size for simulated samples

## nitems <- 12 # number of items per scale

## noptions <- 4 # number of response options per item

## ## Read in the data

## org <- read.table(file.choose(), header=T, sep=",")

## # Read: "SDQ-12 (longitudinal blinded groups for QLR Special Issue).csv"

## data.frame(names(org))

## ggplot(org, aes(x=T1_Sum_Score)) +

## geom_histogram(binwidth=1, colour="black", fill="white") +

## dev.new(width=5, height=4)

## ggplot(org, aes(x=T2_Sum_Score)) +

## geom_histogram(binwidth=1, colour="black", fill="white") +

## dev.new(width=5, height=4)

## ggplot(org, aes(x=T3_Sum_Score)) +

## geom_histogram(binwidth=1, colour="black", fill="white") +

## dev.new(width=5, height=4)

## ggplot(org, aes(x=T4_Sum_Score)) +

## geom_histogram(binwidth=1, colour="black", fill="white") +

## dev.new(width=5, height=4)

## ## Reverse code item scores

## table(org[,5])

## org[, 5:52] <- 3-org[, 5:52]

## ## Set aside the PGIC scores

## pgic <- org[,2:4]

## # head(pgic)

## ## Recode and dichotomize TR items

## table(org$PGIC_T2)

## org$PGIC_T2[org$PGIC_T2=="Much Improved"] <- 1

## org$PGIC_T2[org$PGIC_T2=="Moderately Improved"] <- 1

## org$PGIC_T2[org$PGIC_T2=="Minimally Improved"] <- 1

## org$PGIC_T2[org$PGIC_T2=="No Change"] <- 0

## org$PGIC_T2[org$PGIC_T2=="Minimally Worse"] <- 0

## org$PGIC_T2[org$PGIC_T2=="Moderately Worse"] <- 0

## org$PGIC_T2[org$PGIC_T2=="Much Worse"] <- 0

## org$PGIC_T2 <- as.numeric(org$PGIC_T2)

## table(org$PGIC_T2)

## mean(org$PGIC_T2) # proportion improved T2

## org$PGIC_T3[org$PGIC_T3=="Much Improved"] <- 1

## org$PGIC_T3[org$PGIC_T3=="Moderately Improved"] <- 1

## org$PGIC_T3[org$PGIC_T3=="Minimally Improved"] <- 1

## org$PGIC_T3[org$PGIC_T3=="No Change"] <- 0

## org$PGIC_T3[org$PGIC_T3=="Minimally Worse"] <- 0

## org$PGIC_T3[org$PGIC_T3=="Moderately Worse"] <- 0

## org$PGIC_T3[org$PGIC_T3=="Much Worse"] <- 0

## org$PGIC_T3 <- as.numeric(org$PGIC_T3)

## table(org$PGIC_T3)

## mean(org$PGIC_T3) # proportion improved T3

## org$PGIC_T4[org$PGIC_T4=="Much Improved"] <- 1

## org$PGIC_T4[org$PGIC_T4=="Moderately Improved"] <- 1

## org$PGIC_T4[org$PGIC_T4=="Minimally Improved"] <- 1

## org$PGIC_T4[org$PGIC_T4=="No Change"] <- 0

## org$PGIC_T4[org$PGIC_T4=="Minimally Worse"] <- 0

## org$PGIC_T4[org$PGIC_T4=="Moderately Worse"] <- 0

## org$PGIC_T4[org$PGIC_T4=="Much Worse"] <- 0

## org$PGIC_T4 <- as.numeric(org$PGIC_T4)

## table(org$PGIC_T4)

## mean(org$PGIC_T4) # proportion improved T4

## ## Select datasets for analyses

## org2 <- org[,c(5:16,17:28,2)] # T1 and T2

## org3 <- org[,c(5:16,29:40,3)] # T1 and T3

## org4 <- org[,c(5:16,41:52,4)] # T1 and T4

## ## Add the PGIC ratings

## org2 <- data.frame(org2, pgic$PGIC_T2)

## org3 <- data.frame(org3, pgic$PGIC_T3)

## org4 <- data.frame(org4, pgic$PGIC_T4)

## cbind(names(org2[1:12]),names(org2[13:24]))

## names(org2[25])

## names(org2[26])

## cbind(names(org3[1:12]),names(org3[13:24]))

## names(org3[25])

## names(org3[26])

## cbind(names(org4[1:12]),names(org4[13:24]))

## names(org4[25])

## names(org4[26])

## ###################################

## ## Start with one dataset

## dat <- org2[,1:25] ### CHOOSE ORG2, ORG3 OR ORG4

## gpc <- org2[,26] ### CHOOSE ORG2, ORG3 OR ORG4

## ## Simplify item names

## names(dat)[1:12] <- paste0('v1', '_', 1:12)

## names(dat)[13:24] <- paste0('v2', '_', 1:12)

## names(dat)[25] <- "trt"

## ## Longitudinal IRT

## itemloadings <- rep(1:nitems, times = 2)

## itemloadings <- c(itemloadings, NA)

## model <- 'Time1 = 1-12, 25

## Time2 = 13-24, 25

## COV = Time2*Time2, Time1*Time2

## MEAN = Time2'

## # construct constraints dynamically

## # obtain starting values

## sv <- bfactor(dat, itemloadings, model, pars='values')

## # set up within time constraints

## wtconstr <- sv$parnum[(sv$name == 'a1' | sv$name == 'a2') & sv$est]

## # create constraint list

## constraints <- list()

## itemnames <- colnames(dat)

## pick <- c(0, nitems)

## for(i in 1:nitems){

## # accross time item constraints

## constraints[[paste0('slope.', i)]] <- sv$parnum[sv$name == paste0('a',2+i) & sv$est]

## for(j in 1:(noptions-1)){

## constraints[[paste0('intercept.', i, '_', j)]] <-

## sv$parnum[sv$name == paste0('d',j) & (sv$item %in% itemnames[pick + i]) & sv$est]

## }

## #across time constraints

## constraints[[paste0('Time.', i)]] <- wtconstr[pick + i]

## }

## ( mod <- bfactor(dat, itemloadings, model, constrain=constraints, TOL=1e-3,

## itemtype = 'graded', optimizer = 'nlminb') )

## # Determine item fit

## itemfit(mod, QMC=TRUE)

## ## MIC in terms of theta change

## cf <- coef(mod, simplify=TRUE)

## cf

## ( MIC.theta <- -cf$items[2*nitems+1,nitems+3]/cf$items[2*nitems+1,2] )

## ##### MIC in terms of scale score change

## ## Simulation method using the parameters of model

## a <- cf$items[,1:(nitems+2)]

## a

## d <- cf$items[,(nitems+3):(nitems+5)]

## d

## th1 <- rnorm(N.mic)

## th2 <- th1 + MIC.theta

## th3 <- rnorm(N.mic)

## th4 <- rnorm(N.mic)

## th5 <- rnorm(N.mic)

## th6 <- rnorm(N.mic)

## th7 <- rnorm(N.mic)

## th8 <- rnorm(N.mic)

## th9 <- rnorm(N.mic)

## th10 <- rnorm(N.mic)

## th11 <- rnorm(N.mic)

## th12 <- rnorm(N.mic)

## th13 <- rnorm(N.mic)

## th14 <- rnorm(N.mic)

## Theta <- data.frame(th1,th2,th3,th4,th5,th6,th7,th8,th9,

## th10,th11,th12,th13,th14)

## Theta <- as.matrix(Theta)

## dat1 <- simdata(a,d,itemtype='graded',Theta=Theta)

## dat1 <- as.data.frame(dat1)

## sum1 <- rowSums(dat1[,1:nitems])

## ggplot(dat1, aes(x=sum1)) +

## geom_histogram(binwidth=1, colour="black", fill="white") +

## dev.new(width=5, height=4)

## sum2 <- rowSums(dat1[,(nitems+1):(2*nitems)])

## ggplot(dat1, aes(x=sum2)) +

## geom_histogram(binwidth=1, colour="black", fill="white") +

## dev.new(width=5, height=4)

## sumdif <- sum2 - sum1

## ggplot(dat1, aes(x=sumdif)) +

## geom_histogram(binwidth=1, colour="black", fill="white") +

## dev.new(width=5, height=4)

## ( mic.sy <-

## median(sumdif) ) # MIC based on medium difference score

## ( mic.sx <-

## mean(sumdif) ) # MIC based on mean difference score

## ###### Model without accounting for local dependence

## model <- 'Time1 = 1-12, 25

## Time2 = 13-24, 25

## COV = Time2*Time2, Time1*Time2 # Variance Time2 and covariance T1-T2 free

## MEAN = Time2'

## # construct constraints dynatically

## # obtain starting values

## sv <- mirt(dat, model, pars='values')

## # sv[,1:9]

## # set up within time constraints

## wtconstr <- sv$parnum[(sv$name == 'a1' | sv$name == 'a2') & sv$est]

## # wtconstr

## # create constraint list

## constraints <- list()

## itemnames <- colnames(dat)

## pick <- c(0, nitems)

## for(i in 1:nitems){

## # accross time item constraints

## # constraints[[paste0('slope.', i)]] <- sv$parnum[sv$name == paste0('a',2+i) & sv$est]

## for(j in 1:(noptions-1)){

## constraints[[paste0('intercept.', i, '_', j)]] <-

## sv$parnum[sv$name == paste0('d',j) & (sv$item %in% itemnames[pick + i]) & sv$est]

## }

## # across time constraints

## constraints[[paste0('Time.', i)]] <- wtconstr[pick + i]

## }

## moda <- mirt(dat, model, constrain=constraints, TOL=1e-3,

## itemtype = 'graded', optimizer = 'nlminb')

## # Determine item fit

## itemfit(moda)

## # MIC in terms of theta change

## ( cf <- coef(moda, simplify=TRUE)$items ) # Variance-covariance matrix

## ( mic.theta <- -cf[2*nitems+1,3]/cf[2*nitems+1,2] )

## ## Compare models

## M2(mod, QMC=TRUE) # model with accounting for LD

## M2(moda, QMC=TRUE) # model without accounting for LD

## anova(mod, moda) # The simpler model appears not to be sign. worse

## ##### MIC in terms of scale score change

## ## Simulation method using the modelled parameters

## ( a <- cf[,1:2] )

## ( d <- cf[,3:5] )

## th1 <- rnorm(N.mic)

## th2 <- th1 + mic.theta

## Theta <- data.frame(th1,th2)

## Theta <- as.matrix(Theta)

## dat1 <- simdata(a,d,itemtype='graded',Theta=Theta)

## dat1 <- as.data.frame(dat1)

## sum1 <- rowSums(dat1[,1:nitems])

## ggplot(dat1, aes(x=sum1)) +

## geom_histogram(binwidth=1, colour="black", fill="white") +

## dev.new(width=5, height=4)

## sum2 <- rowSums(dat1[,(nitems+1):(2*nitems)])

## ggplot(dat1, aes(x=sum2)) +

## geom_histogram(binwidth=1, colour="black", fill="white") +

## dev.new(width=5, height=4)

## sumdif <- sum2 - sum1

## ggplot(dat1, aes(x=sumdif)) +

## geom_histogram(binwidth=1, colour="black", fill="white") +

## dev.new(width=5, height=4)

## ( mic.sy <-

## median(sumdif) ) # MIC based on medium difference score: 4

## ( mic.sx <-

## mean(sumdif) ) # MIC based on mean difference score: 3.9

## #############################

## ## ROC analysis

## dat$xo1 <- rowSums(dat[,1:nitems]) # Sumscore T1

## dat$xo2 <- rowSums(dat[,(nitems+1):(2*nitems)]) # Sumscore T2

## dat$xoc <- dat$xo2 - dat$xo1 # Change score

## rocobj <- roc(dat$trt, dat$xoc, quiet = TRUE)

## mic.roc <- coords(rocobj, x="best", input="threshold", ret="threshold",

## best.method="closest.topleft", transpose = TRUE)

## mic.roc[sample(length(mic.roc),1)] # ROC-based MIC: 1.5

## ## Logistic regression

## ( q <- mean(dat$trt) ) # proportion improved

## ( Cor <- cor(dat$trt,dat$xoc) )

## ( sd.tdif <- sd(dat$xoc) )

## mylogit <- glm(trt ~ xoc, data = dat, family = "binomial")

## C <- coef(mylogit)[1] # intercept coefficient C

## B <- coef(mylogit)[2] # regression coefficient B

## p <- log(q/(1-q)) # p = logodds(pre)

## ( mic.pred <- (p-C)/B ) # MIC(predicted)

## cf <- 0.09 * sd.tdif + 0.103 * sd.tdif * Cor

## ( mic.adj <- mic.pred - cf * p ) # MIC(adjusted)

## ## Mean change MIC

## table(gpc)

## # Proportion minimally improved

## length(gpc[gpc=="Minimally Improved"])/dim(dat)[1]

## # MIC mean change

## mean(dat$xoc[gpc=="Minimally Improved"])

## # Boxplots of change score by PGIC category

## dat$gpc <- gpc

## dat$gpc[gpc=="Much Improved"] <- 7

## dat$gpc[gpc=="Moderately Improved"] <- 6

## dat$gpc[gpc=="Minimally Improved"] <- 5

## dat$gpc[gpc=="No Change"] <- 4

## dat$gpc[gpc=="Minimally Worse"] <- 3

## dat$gpc[gpc=="Moderately Worse"] <- 2

## dat$gpc[gpc=="Much Worse"] <- 1

## dat$gpc <- as.factor(dat$gpc)

## ggplot(dat,

## aes(x=gpc, y=xoc)) +

## geom_boxplot(notch=F) +

## dev.new(width=5, height=4)

## #####################################################################

## #####################################################################

## ####### BOOTSTRAPPEN

## nboot <- 1000 # number of bootstrap samples

## mic.roc <- numeric(nboot)

## mic.pred <- numeric(nboot)

## mic.adj <- numeric(nboot) # old adjusted MIC (2017)

## boot <- data.frame(mic.roc, mic.pred, mic.adj)

## boot$prop.imp <- numeric(nboot) # proportion improved

## boot$mic.mean <- numeric(nboot) # mean change MIC

## boot$mic.theta <- numeric(nboot) # LIRT MIC in terms of theta change

## boot$mic.sy <- numeric(nboot) # LIRT MIC in terms of scale score change, median

## boot$mic.sx <- numeric(nboot) # LIRT MIC in terms of scale score change, mean

## org <- org2 ### @@@ BEWARE TO CHANGE org2/org3/org4 !!!

## ## Simplify item names

## names(org)[1:12] <- paste0('v1', '_', 1:12)

## names(org)[13:24] <- paste0('v2', '_', 1:12)

## names(org)[25] <- "trt"

## data.frame(names(org))

## ### START BOOTSTRAP

## start.time <- Sys.time()

## for(k in 1:nboot) {

## print(k)

## dat <- org[sample(1:dim(org)[1], dim(org)[1], replace=TRUE),]

## gpc <- dat[,26]

## dat <- dat[,1:25]

## ## Longitudinal IRT

## model <- 'Time1 = 1-12, 25

## Time2 = 13-24, 25

## COV = Time2*Time2, Time1*Time2 # Variance Time2 and covariance T1-T2 free

## MEAN = Time2'

## # construct constraints dynatically

## # obtain starting values

## sv <- mirt(dat, model, pars='values')

## # set up within time constraints

## wtconstr <- sv$parnum[(sv$name == 'a1' | sv$name == 'a2') & sv$est]

## # wtconstr

## # create constraint list

## constraints <- list()

## itemnames <- colnames(dat)

## pick <- c(0, nitems)

## for(i in 1:nitems){

## # accross time item constraints

## # constraints[[paste0('slope.', i)]] <- sv$parnum[sv$name == paste0('a',2+i) & sv$est]

## for(j in 1:(noptions-1)){

## constraints[[paste0('intercept.', i, '_', j)]] <-

## sv$parnum[sv$name == paste0('d',j) & (sv$item %in% itemnames[pick + i]) & sv$est]

## }

## # across time constraints

## constraints[[paste0('Time.', i)]] <- wtconstr[pick + i]

## }

## cap <- capture.output( mod <- mirt(dat, model, constrain=constraints, TOL=1e-3,

## itemtype = 'graded', optimizer = 'nlminb') )

## ( cf <- coef(mod, simplify=TRUE)$items ) # Variance-covariance matrix

## ( boot$mic.theta[k] <-

## mic.theta <- -cf[2*nitems+1,3]/cf[2*nitems+1,2] )

## ##### MIC in terms of scale score change

## ## Simulation method

## ( a <- cf[,1:2] )

## ( d <- cf[,3:5] )

## th1 <- rnorm(N.mic)

## th2 <- th1 + mic.theta

## Theta <- data.frame(th1,th2)

## Theta <- as.matrix(Theta)

## dat1 <- simdata(a,d,itemtype='graded',Theta=Theta)

## sum1 <- rowSums(dat1[,1:nitems])

## # hist(sum1)

## sum2 <- rowSums(dat1[,(nitems+1):(2*nitems)])

## # hist(sum2)

## sumdif <- sum2 - sum1

## # hist(sumdif) # The distribution of sumdif is normal

## ( boot$mic.sy[k] <-

## median(sumdif) ) # MIC based on medium difference score

## ( boot$mic.sx[k] <-

## mean(sumdif) ) # MIC based on mean difference score

## ##### ROC analysis

## dat$xo1 <- rowSums(dat[,1:nitems]) # Sumscore T1

## dat$xo2 <- rowSums(dat[,(nitems+1):(2*nitems)]) # Sumscore T2

## dat$xoc <- dat$xo2 - dat$xo1 # Change score

## rocobj <- roc(dat$trt, dat$xoc, quiet = TRUE)

## mic.roc <- coords(rocobj, x="best", input="threshold", ret="threshold",

## best.method="closest.topleft", transpose = TRUE)

## ( boot$mic.roc[k] <- mic.roc[sample(length(mic.roc),1)] ) # ROC-based MIC

## ## Logistic regression

## ( boot$prop.imp[k] <- q <- mean(dat$trt) )

## ( Cor <- cor(dat$trt,dat$xoc) )

## ( sd.tdif <- sd(dat$xoc) )

## mylogit <- glm(trt ~ xoc, data = dat, family = "binomial")

## C <- coef(mylogit)[1] # intercept coefficient C

## B <- coef(mylogit)[2] # regression coefficient B

## p <- log(q/(1-q)) # p = logodds(pre)

## ( boot$mic.pred[k] <- mic.pred <- (p-C)/B ) # MIC(predicted)

## cf <- 0.09 * sd.tdif + 0.103 * sd.tdif * Cor

## ( boot$mic.adj[k] <- mic.pred - cf * p ) # MIC(adjusted)

## ## Mean change MIC

## ( boot$mic.mean[k] <- mean(dat$xoc[gpc=="Minimally Improved"]) ) # MIC(mean change)

## }

## ## Save bootstrap results

## write.table(boot, file = "E:/boot-res-T4.dat", sep = " ", row.names = F, col.names = T)

## end.time <- Sys.time()

## time.taken <- end.time - start.time

## time.taken

## ########### END OF BOOTSTRAP

## fix(boot)

## data.frame(names(boot))

## round(mean(boot$prop.imp),2)

## round(mean(boot$mic.theta),2)

## round(mean(boot$mic.mean),2)

## round(mean(boot$mic.roc),2)

## round(mean(boot$mic.pred),2)

## round(mean(boot$mic.adj),2)

## round(mean(boot$mic.sy),2)

## round(mean(boot$mic.sx),2)

## round(quantile(boot$prop.imp, c(0.025, 0.975)),2)

## round(quantile(boot$mic.theta, c(0.025, 0.975)),2)

## round(quantile(boot$mic.mean, c(0.025, 0.975)),2)

## round(quantile(boot$mic.roc, c(0.025, 0.975)),2)

## round(quantile(boot$mic.pred, c(0.025, 0.975)),2)

## round(quantile(boot$mic.adj, c(0.025, 0.975)),2)

## round(quantile(boot$mic.sy, c(0.025, 0.975)),2)

## round(quantile(boot$mic.sx, c(0.025, 0.975)),2)

## 4. Comparison of models for dichotomous and ordinal categorical transition ratings

A graded response IRT model for an ordinal categorical TR item would be

$ln\left( \frac{P\left( {TR}_{j}\geq c \right)}{P\left( {TR}_{j}<c \right)} \right)=\alpha_{TR}\left( {d\theta}_{j}-\beta_{{TR}_{c}} \right)$

where *c* indicates the relevant category of the TR item. In the data example, the TR item originally had seven categories which can be numbered 0: Much worse, 1: Moderately worse, 2: Minimally worse, 3: No change, 4: Minimally improved, 5: Moderately improved, 6: Much improved. Thus, six threshold parameters will be estimated for the ordinal categorical TR item. To compare with the dichotomous analyses, the relevant category is 4. In Mplus, the only necessary change in model specification would be

[ANCHOR$4] (Lambda); !!! Identifying the correct lambda for an ordinal categorial TR item;

instead of

[ANCHOR$1] (Lambda); !!! Identifying the lambda for a dichotomous TR item;

of course, the data set needs to include the ordinal categorical version of the TR item.

The table below compares item parameter estimates based on the dichotomous and ordinal categorical TR item. For follow-up 1 and 2 the estimates are identical, but with slightly lower confidence intervals for the ordinal item. For follow-up 3, estimates are close, but the estimate from the ordinal item is closer to the true value of 0.50.

| **Item parameter estimates for a dichotomous and ordinal categorical TR item** | | | | | | | | |
| --- | --- | --- | --- | --- | --- | --- | --- | --- |
|  | Follow-up 1 | |  | Follow-up 2 | |  | Follow-up 3 | |
|  | Est | (95% CI) |  | Est | (95% CI) |  | Est | (95% CI) |
| $\beta_{TR}$ dichotomous | 0.56 | (0.48 : 0.65) |  | 0.50 | (0.39 : 0.61) |  | 0.46 | (0.31 : 0.61) |
| $\beta_{{TR}_{4}}$ ordinal 7 categories | 0.56 | (0.48 : 0.65) |  | 0.50 | (0.41 : 0.60) |  | 0.51 | (0.36 : 0.65) |
